# P-1482. Ceftazidime-Avibactam for Treating Bacteremic and Non-Bacteremic Carbapenemase-Producing Enterobacterales Infections in Immunocompromised Patients

**DOI:** 10.1093/ofid/ofae631.1652

**Published:** 2025-01-29

**Authors:** Fabián Herrera, María V Leone, Diego Torres, Rocío Rojas, Florencia Bues, Andrés N Rearte, Elena Temporiti, Marcia Querci, Jimena Carlos, Federico Nicola, Silvia Relloso, Jorgelina Smayevsky, Pablo E Bonvehi

**Affiliations:** Centro de Educación Médica e Investigaciones Clínicas, CEMIC, BUENOS AIRES, Ciudad Autonoma de Buenos Aires, Argentina; Hospital Universitario CEMIC, Buenos Aires, Ciudad Autonoma de Buenos Aires, Argentina; Centro de Educación Médica e Investigaciones Clínicas, CEMIC, BUENOS AIRES, Ciudad Autonoma de Buenos Aires, Argentina; Hospital Universitario CEMIC, Buenos Aires, Ciudad Autonoma de Buenos Aires, Argentina; Hospital Universitario CEMIC, Buenos Aires, Ciudad Autonoma de Buenos Aires, Argentina; Vicente Lopez Municipality, Buenos Aires, Ciudad Autonoma de Buenos Aires, Argentina; Hospital Universitario CEMIC, Buenos Aires, Ciudad Autonoma de Buenos Aires, Argentina; Hospital Universitario CEMIC, Buenos Aires, Ciudad Autonoma de Buenos Aires, Argentina; Hospital Universitario CEMIC, Buenos Aires, Ciudad Autonoma de Buenos Aires, Argentina; Hospital Universitario CEMIC, Buenos Aires, Ciudad Autonoma de Buenos Aires, Argentina; Hospital Universitario CEMIC, Buenos Aires, Ciudad Autonoma de Buenos Aires, Argentina; Hospital Universitario CEMIC, Buenos Aires, Ciudad Autonoma de Buenos Aires, Argentina; Hospital Universitario CEMIC, Buenos Aires, Ciudad Autonoma de Buenos Aires, Argentina

## Abstract

**Background:**

Ceftazidime-avibactam (CA) and CA plus aztreonam (ATM) are the preferred treatment options for KPC and metallo-beta-lactamase (MBL) carbapenemase-producing Enterobacterales infections (KPC-CPE and MBL-CPE), respectively. There are not many studies in immunocompromised patients (IP).

**Methods:**

Observational and prospective cohort study. All episodes of KPC-CPE and MBL-CPE infections in IP admitted to a university hospital from August 2018 to April 2023, who received definitive antibiotic therapy (DAT) with CA or CA+ATM for at least 48 hours, were included. Non-bacteremic episodes (NBE) were compared with those with bacteremia (BE). Logistic regression analysis was performed to identify risk factors for 30-day mortality (RFM).

**Results:**

Seventy-six episodes were included (36 NBE and 40 BE). Hematological malignancies and neutropenia were more frequent in BE (NBE 13.9% vs. BE 50.2%, p< 0.001; and NBE 5.6% vs. BE 42.5%, p< 0.001), while solid organ transplantation was in NBE (NBE 66.7% vs. BE 32.5%, p=0.003). The urinary source was more frequent in NBE (NBE 72.2% vs. BE 30%, p< 0.001), while the abdominal source was in BE (NBE 2.8% vs. BE 17.5%, p=0.04). *K. pneumoniae* was the main isolated microorganism, and KPC-CPE was the most common resistance mechanism in both groups, followed by MBL-CPE, without significant differences. Septic shock at onset was higher in BE (NBE 8.3% vs. BE 30%, p=0.18). Empirical treatment was appropriate in 59.3% of NBE vs. 75% of BE, p=0.12. Seven-day mortality, 30-day overall and infection-related mortality in NBE and BE were 2.8% vs. 5%, p=0.40; 11.1% vs. 27.5%, p=0.07; and 0% vs. 10%, p=0.07; respectively. The RFM were APACHE II score >20 [OR 5.80 (95% CI 1.19-28.17)], and acute renal failure [OR 16.15 (95% CI 1.78-146)].

**Conclusion:**

KPC-CPE and MBL-CPE infections in IP with or without bacteremia who received DAT with CA or CA+ATM had low early and 30-day infection-related mortality. RFM were related to the severity and complications of the infections.

**Disclosures:**

**Diego Torres, Medical Doctor**, Gador: Honoraria|GSK: Advisor/Consultant|Khight Therapeutics: Honoraria|MSD: Honoraria

